# Optimization of Ultraviolet-B Treatment for Enrichment of Total Flavonoids in Buckwheat Sprouts Using Response Surface Methodology and Study on Its Metabolic Mechanism

**DOI:** 10.3390/foods13233928

**Published:** 2024-12-05

**Authors:** Jiyuan Xue, Meixia Hu, Jia Yang, Weiming Fang, Yongqi Yin

**Affiliations:** 1 College of Food Science and Engineering, Yangzhou University, Yangzhou 210095, China; mz120232144@stu.yzu.edu.cn (J.X.); mz120222068@stu.yzu.edu.cn (M.H.); wmfang@yzu.edu.cn (W.F.); 2 Yangzhou Center for Food and Drug Control, Yangzhou 225000, China; jiajia82112001@163.com

**Keywords:** buckwheat, ultraviolet-B, sprouts, flavonoids, response surface methodology

## Abstract

Buckwheat possesses significant nutritional content and contains different bioactive compounds, such as total flavonoids, which enhance its appeal to consumers. This study employed single-factor experiments and the response surface methodology to identify the optimal germination conditions for enhancing the total flavonoid content in buckwheat sprouts through ultraviolet-B treatment. The research showed that buckwheat sprouts germinated for 3 days at a temperature of 28.7 °C while being exposed to ultraviolet-B radiation at an intensity of 30.0 μmol·m^−2^·s^−1^ for 7.6 h per day during the germination period resulted in the highest total flavonoid content of 1872.84 μg/g fresh weight. Under these specified conditions, ultraviolet-B treatment significantly elevated the activity and gene expression levels of enzymes related to the phenylpropanoid metabolic pathway, including phenylalanine ammonia-lyase, cinnamic acid 4-hydroxylase, 4-coumarate coenzyme A ligase, and chalcone isomerase. Ultraviolet-B treatment caused oxidative damage to buckwheat sprouts and inhibited their growth, but ultraviolet-B treatment also enhanced the activity of key enzymes in the antioxidant system, such as catalase, peroxidase, superoxide dismutase, and ascorbate peroxidase. This research provided a technical reference and theoretical support for enhancing the isoflavone content in buckwheat sprouts through ultraviolet-B treatment.

## 1. Introduction

Sprouts have been an important component of human diets since ancient times. In recent years, with increasing attention to nutritious and healthy foods, seed germination has become an inexpensive method to enhance the bioavailability and nutritional composition of grains and legumes [1]. The germination process induces a series of biochemical changes in seeds: under the action of endogenous enzymes, starch, proteins, and fats are mobilized or hydrolyzed, while harmful and anti-nutritional substances, including phytic acid, protease inhibitors, and tannins, are degraded [2,3]. For example, germinated brown rice, as a typical representative of sprouted grains, showed a significant accumulation of γ-amino butyric acid (GABA) and enhanced antioxidant capacity [4]. To enhance the concentration of significant bioactive compounds in sprouts, an increasing number of studies have utilized external physical or chemical stimulation during plant germination [2]. Treatments such as ultraviolet light [5,6], light-emitting diodes [7], microwaves [8,9], magnetic fields [10], and plant hormones [11] have been applied to the germination process of many plants to further enhance the nutritional value of sprouts. 

Buckwheat is a crop that predominantly thrives under severe environmental conditions, such as elevated ultraviolet radiation and drought. It is distinguished by its gluten-free nature and significant flavonoid content [12]. Interest in the use and development of buckwheat raw materials is increasing. Nevertheless, the existence of anti-nutritional components as protease inhibitors and tannins in buckwheat leads to reduced protein digestibility (79.9%) and the diminished bioavailability of beneficial compounds [13]. In recent years, sprouted buckwheat has become a new raw material for producing functional foods, with buckwheat sprouts being used as edible vegetables or as natural food colorants. More importantly, the content of anti-nutritional substances in sprouted buckwheat decreases or even disappears, the amino acid composition becomes more balanced, and the digestibility and bioavailability of nutrients improve, especially under specific germination conditions that further promote the accumulation of flavonoids [14,15,16]. Flavonoids in plants are well known for their nutritional value [17,18]. The flavonoids in buckwheat mainly include flavonols, flavones, and anthocyanins [19]. Multiple studies have demonstrated that ultraviolet irradiation significantly affects the flavonoid concentration in buckwheat sprouts. Ultraviolet (UV) irradiation markedly enhanced the levels of flavonoids in buckwheat sprouts [20]. Ultraviolet-B (UV-B) in conjunction with blue light treatment and Ultraviolet-A/Ultraviolet-C treatment considerably augmented the total flavonoid content by enhancing the activity of crucial enzymes involved in flavonoid production and elevating the expression levels of associated genes in buckwheat sprouts [21,22]. The activity of major enzymes involved in flavonoid production in buckwheat sprouts exhibited a substantial positive correlation with the total flavonoid concentration [22,23,24]. Yao et al. [25] found significant differences in total flavonoid accumulation among 15 buckwheat varieties grown under different natural UV-B irradiation levels. The total flavonoid content in four Polish buckwheat varieties showed completely different trends under the same UV-B treatment [26]. The investigations demonstrated that the concentration of flavonoid chemicals in buckwheat sprouts is affected by the intensity and duration of ultraviolet irradiation, in addition to the specific buckwheat type. Therefore, it is necessary to find suitable germination processes when using ultraviolet treatment to promote the accumulation of flavonoid compounds.

Individuals are concentrating on the ideal amalgamation of germination conditions (external treatments, germination temperature, and germination time) to achieve superior quality sprouts, particularly to enhance the biosynthesis of bioactive compounds (such as GABA, flavonoids, total phenols, etc.). These objectives are accomplished by the application of intricate statistical methodologies, particularly the response surface methodology (RSM). Le et al. [27] utilized RSM to adjust the soaking duration, pH, germination period, and germination temperature of brown rice to maximize anthocyanin content in germinated brown rice. Zhang et al. [28] maximized the total flavonoid content in Finger Millet sprouts by optimizing the germination temperature, germination time, and light exposure time. The ideal conditions for resveratrol accumulation were as follows: Peanuts subjected to 0.8 mM phenylalanine and 30 minutes of ultrasonic treatment at 35 °C and 240 W exhibited a resveratrol level in sprouts that was 9.4 times greater than that of non-germinated peanuts [29]. Prior research has investigated the influence of germination temperature and duration on flavonoid chemicals during the buckwheat malting process, determining that germination temperature has a more significant effect than germination duration [30].

So far, few studies have systematically optimized the germination conditions for flavonoid enrichment in buckwheat sprouts, especially the optimization of germination conditions with UV treatment using the response surface methodology. Meanwhile, Pintian 2 is a new buckwheat variety in China. Therefore, we conducted in-depth research on Pintian No. 2 in this direction. This study thoroughly investigated how UV-B irradiation intensity, exposure duration, germination temperature, and germination time affect the total flavonoid content in buckwheat sprouts. Using single-factor and response surface methodologies, the ideal germination conditions were optimized to enhance flavonoid biosynthesis. Under optimal UV-B treatment, the physiological and biochemical characteristics, antioxidant capacity, and activity and gene expression of key enzymes involved in flavonoid biosynthesis and antioxidant pathways in buckwheat sprouts were systematically analyzed. This research provides a foundation for understanding the production and regulatory mechanisms of flavonoid-enriched buckwheat sprouts.

## 2. Materials and Methods

### 2.1. Materials Treatment and Experimental Design

Seeds for the experiment, Pintian No. 2, were provided by the Shanxi Academy of Agricultural Sciences in 2022. Before germination, they were stored at −18 °C. A sufficient batch was sterilized in a 1% (*v*/*v*) sodium hypochlorite solution for 10 min, followed by rinsing with deionized water to achieve a neutral pH. Afterward, the seeds were soaked at 25 °C for 12 h to initiate germination. Thereafter, the seeds experienced an uninterrupted germination process. The optimal conditions for germination were further established through single-factor experiments and RSM tests. The sprouts were sampled to assess physiological and biochemical indicators, including total flavonoid content, total phenol content, metabolic enzyme activity, antioxidant enzyme activity, antioxidant capacity, and gene expression.

### 2.2. RSM Methods for Determining Optimal Germination Conditions

Seeds were positioned in germination boxes at varying temperatures and subjected to varying UV-B treatment intensities for different durations, and samples were taken at different germination times. Subsequent to the determination of initial parameters for germination time, temperature, duration, and intensity of UV-B treatment via single-factor tests, we refined the germination conditions to enhance the total flavonoid content in buckwheat sprouts. For more details, refer to Supplementary Tables S1 and S2.

### 2.3. Total Flavonoids and Phenols Content

Total phenols were determined according to Kan et al. [31]. Specifically, sprouts were subjected to extraction using 50% (*v*/*v*) methanol. The extraction mixture was then centrifuged at a force of 10,000× *g* for 15 min to separate the soluble compounds. The resulting supernatant, measuring 1 mL, was carefully combined with 1.0 mL of 0.2 mM Folin-phenol reagent and 2.0 mL of 2% (*w*/*v*) sodium carbonate. This mixture was allowed to react for 2 h in darkness to prevent photo-degradation. The total phenolic content was subsequently quantified by measuring the absorbance at a wavelength of 765 nm with a UV spectrophotometer (DR6000, Shanghai Ruishi Technology Co., Shanghai, China).

Flavonoid levels were assessed following Yin et al. [32], with rutin as the calibration reference. A methanolic extract was combined with a 2% aluminum chloride solution (1:1, *v*/*v*) and left to react at room temperature for 15 min. Absorbance at 430 nm was recorded using a UV spectrophotometer, and the total flavonoid content was calculated in μg of rutin, referencing identically processed rutin standards.

### 2.4. H_2_O_2_, MDA, and $O_{2}^{-.}$—Content

The malondialdehyde (MDA) and hydrogen peroxide (H_2_O_2_) contents were determined by Yin et al. [32]. Buckwheat sprouts were homogenized with trichloroacetic acid (TCA) and centrifuged at 8000× *g* for 15 min. The resulting supernatant was combined with 0.67% thiobarbituric acid (1:1, *v*/*v*), and the absorbance was recorded using a UV spectrophotometer (DR6000, Shanghai Ruishi Technology Co., Shanghai, China) to determine MDA content at 450 nm, 532 nm, and 600 nm.

For hydrogen peroxide measurement, buckwheat sprouts were homogenized with PBS buffer and centrifuged at 8000× *g* for 15 min. The supernatant was mixed with deionized water (1:4, *v*/*v*), potassium iodide, and TCA, followed by a 30 min reaction. Absorbance at 390 nm was measured with the same spectrophotometer. A standard curve, created using H_2_O_2_ as a reference, enabled the determination of hydrogen peroxide concentration in the samples.

Superoxide anion concentrations ($O_{2}^{-.}$) were determined following Zhao et al. [33] A 1.0 g sample of sprouts was homogenized in 4 mL of phosphate buffer and centrifuged at 8000× *g* for 10 min. From the supernatant, 1 mL was taken and mixed with 0.9 mL of phosphate buffer (pH 7.8) and 0.1 mL of hydroxylamine hydrochloride, incubated at 25 °C for 25 min, and then treated with p-aminobenzenesulfonic acid and α-naphthylamine. The absorbance of the reaction solution was measured at 530 nm. 

### 2.5. Antioxidant Capacity

Buckwheat sprouts were thoroughly homogenized with 80% methanol and centrifuged at 8000× *g* for 10 min, with the resulting supernatant used for analysis. The 2,2-diphenyl-1-picrylhydrazyl (DPPH) and 2,2′-azino-bis(3-ethylbenzothiazoline-6-sulfonic acid) (ABTS) scavenging rates were determined following the method described by Du et al. [34]. Specifically, 0.1 mL of supernatant was mixed with 2.9 mL of DPPH solution and incubated in the dark for 30 min, and the absorbance at 517 nm was recorded using a UV spectrophotometer (DR6000, Shanghai Ruishi Technology Co., Shanghai, China), with the results expressed as the DPPH scavenging rate (%).

Similarly, 0.1 mL of supernatant was added to 2.9 mL of the ABTS working solution, incubated under the same conditions, and the absorbance at 734 nm was measured using the same UV spectrophotometer, with the results given as the ABTS scavenging rate (%).

The Ferric ion-reducing antioxidant power (FRAP) assay was also performed according to Du et al. [34]. A mixture of 0.25 mL of the supernatant, 1.0 mL of the phosphate buffer, and 1.0 mL of potassium ferricyanide was incubated, followed by the addition of 1.0 mL of 10% trichloroacetic acid with vigorous mixing. Absorbance at 700 nm was measured using the UV spectrophotometer, and the FRAP values were calculated as the scavenging rate (%).

### 2.6. Antioxidant Enzyme Activity

The activities of antioxidant enzymes were determined by Yin et al. [32]. A single unit of activity for both catalase (CAT) and peroxidase (POD) is meticulously defined as a variation or change of 0.02 per minute when measured at optical densities of 240 nm and 470 nm (UV spectrophotometer, DR6000, Shanghai Ruishi Technology Co., Shanghai, China), respectively. A specific unit for measuring the activity of superoxide dismutase (SOD) was established, which is characterized by a variation of 0.02 per minute at optical densities of 560 nm (UV spectrophotometer, DR6000, Shanghai Ruishi Technology Co., Shanghai, China), correspondingly. Ascorbate peroxidase (APX) activity was determined by Bin, et al. [35]

### 2.7. Metabolic Enzyme Activity Assay

The activities of metabolic enzyme activity were determined by Ma et al. [36]. One gram of buckwheat sprouts was blended with 5 mL of a Tris-HCl buffer solution, carefully adjusted to a pH of 8.9 and a concentration of 0.1 M. Following this procedure, the resulting homogenate underwent centrifugation at an acceleration of 12,000× *g* for 15 min, maintained at a temperature of 4 °C. This centrifugation step was conducted to isolate the supernatant, which contains the enzyme activities of interest. For this study, one unit of enzyme activity for Phenylalanine ammonia lyase (PAL), cinnamic acid 4-hydroxylase (C4H), and 4-coumarate coenzyme A ligase (4CL) was defined as a measurable change of 0.01 per minute in optical density readings at 290 nm, 340 nm, and 333 nm with a UV spectrophotometer (DR6000, Shanghai Ruishi Technology Co., Shanghai, China), respectively.

Chalcone isomerase (CHI) activity was conducted using the methodology of Ji et al. [21]. The supernatant derived from the previous extraction operation was employed as a source of crude enzyme. A specific volume of 0.75 mL of the crude enzyme solution was added to a reaction mixture containing 2 mL of Tris-HCl buffer at pH 7.4, 7.5 mg/mL of bovine serum albumin, 50 mM of potassium cyanide, and 50 µL of hydroxylated chalcone solution. The enzymatic reaction was conducted under regulated circumstances at a temperature of 30 °C for 30 min. The activity of CHI was evaluated by monitoring the change in absorbance at a wavelength of 381 nm using a UV spectrophotometer (DR6000, Shanghai Ruishi Technology Co., Shanghai, China).

### 2.8. RNA Extraction and Quantitative Real-Time PCR Analysis

Total RNA was isolated utilizing a Plant RNA Extraction Kit (RC411, Vazyme, Nanjing, China). Total RNA was reverse transcribed following the directions of the PrimeScriptTM RT Master Mix Kit (R423, Vazyme, China). The strip cDNA was quantified in triplicate using the SYBR Green method [37]. Table S3 lists the primers used in this study. The 2^−∆∆Ct^ comparative approach was employed to determine the relative expression levels of genes [38].

### 2.9. Statistical Analysis

This study’s experiments were performed with three biological and three technological replications. The data were expressed as mean ± standard deviation. The data were analyzed via one-way ANOVA, with comparisons conducted using Tukey’s multiple-range test at a significance level of 0.05, employing SPSS software (version 26).

## 3. Results

### 3.1. Effects of Germination Time, UV-B Treatment Time, Germination Temperature, and UV-B Intensity on the Total Flavonoids Content

**Figure 1:**
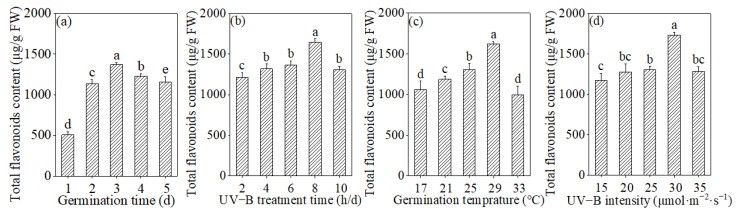
Effects of germination time (**a**), UV-B treatment time (**b**), germination temperature (**c**), and UV-B intensity (**d**) on total flavonoid content. Different lowercase letters represent significant differences between treatment groups.

It is evident from Figure 1 that the four factors—germination time, UV-B treatment time, germination temperature, and UV-B intensity—significantly influence the total flavonoid content in buckwheat sprouts. The total flavonoid content exhibited a trend of an initial increase followed by a subsequent decrease in response to variations in germination time, UV-B treatment time, germination temperature, and intensity of UV-B treatment. Notably, on the third day of germination, when the UV-B treatment time was set at 8 h per day, the germination temperature was maintained at 29 °C, or the UV-B treatment intensity was 30 μmol·m^−2^·s^−1^, the total flavonoid content reached its peak values of 1372.08 μg/g FW, 1645.95 μg/g FW, 1624.88 μg/g FW, and 1735.25 μg/g FW, respectively.

### 3.2. Statistical Analysis of RSM

The findings of the variance analysis, goodness-of-fit assessment, and model adequacy evaluation are encapsulated in Table 1. Utilizing multivariate regression analysis of the experimental data, a quadratic polynomial regression equation was created to represent Y (total flavonoid content) as a function of A (germination time), B (UV-B treatment time), C (germination temperature), and D (UV-B intensity):

*Y* = 1826.00 − 38.44A − 96.46B − 43.34C − 39.84D − 25.16AB + 35.65AC − 62.91AD −        23.07BC − 111.14BD − 20.97CD − 273.44A^2^ − 230.45B^2^ − 270.29C^2^ − 183.27D^2^

**Figure 2:**
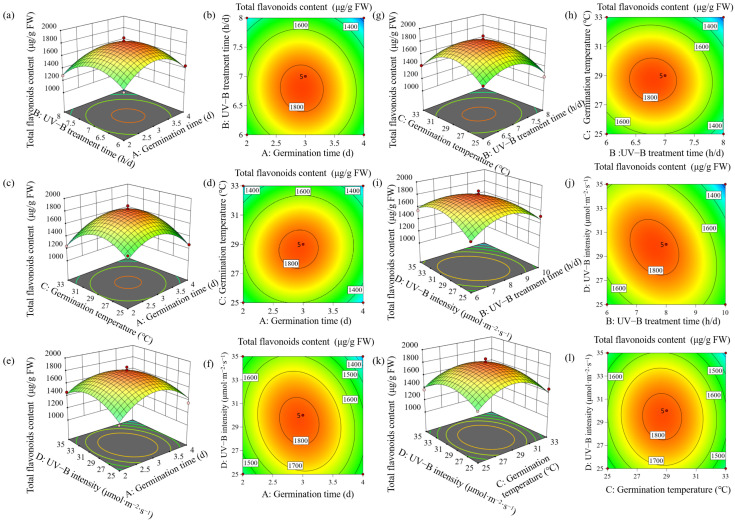
Plots of the interaction of variables on total flavonoid enrichment. Response surface plot of UV−B treatment time and Germination time (**a**), contour map of UV−B treatment time and Germination time (**b**), response surface plot of Germination temperature and Germination time (**c**), contour map of Germination temperature and Germination time (**d**), response surface plot of UV−B intensity and Germination time (**e**), contour map of UV−B intensity and Germination time (**f**), response surface plot of Germination temperature and UV−B treatment time (**g**), contour map of Germination temperature and UV−B treatment time (**h**), response surface plot of UV−B intensity and UV−B treatment time (**i**), contour map of UV−B intensity and UV−B treatment time (**j**), response surface plot of UV−B intensity and Germination temperature (**k**) and contour map of UV−B intensity and Germination temperature (**l**).

It can be seen that the determination coefficient R^2^ (R^2^ = 0.9639) tested the fit of the predictive model (Table 1). The misfit F-value is not statistically significant (*p* > 0.05), thereby affirming the model’s validity. Additionally, the adjusted coefficient of determination (R^2^_adj_ = 0.9278) suggests a high level of significance. The plots illustrating the interaction of each variable on the enrichment of total flavonoids enrichment are presented in Figure 2. The findings indicated that, among the four independent variables examined, UV-B treatment time was the most significant factor. This was followed in importance by germination temperature, UV-B intensity, and germination time. The results indicated that the optimal conditions for enriching total flavonoids in buckwheat sprouts were 3.0 days, UVB treatment time of 7.6 h·d^−1^, germination temperature of 28.7 °C, and UV-B intensity of 30.0 μmol·m^−2^·s^−1^. Under these conditions, the maximum total flavonoid content reached 1872.84 μg/g FW. 

### 3.3. Effects of UV-B Treatment on the Contents of Total Flavonoids and Total Phenols

**Figure 3:**
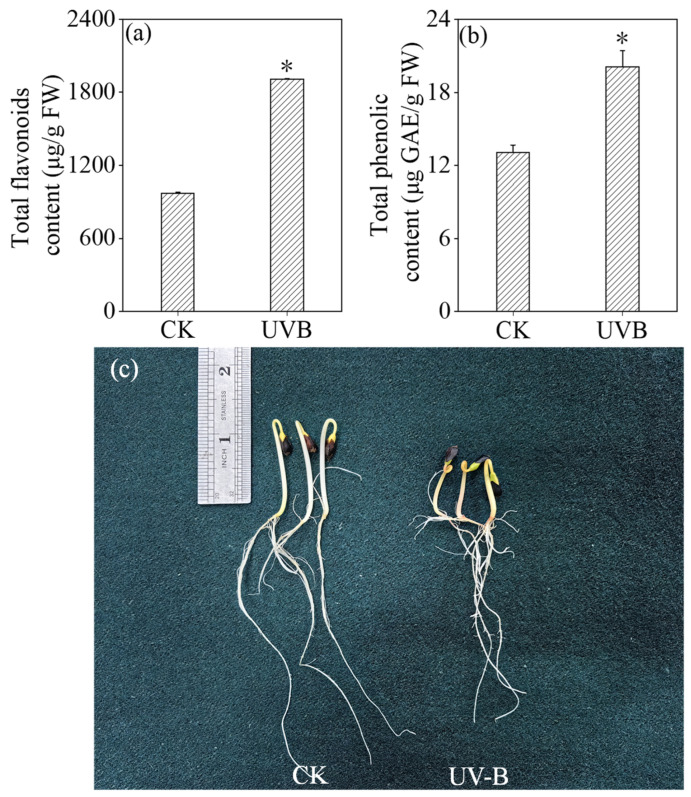
Effects of UV-B treatment on the content of total flavonoid content (**a**), total phenolic (**b**), and growth performance (**c**) of buckwheat sprouts. * Indicates significant differences in indicators among treatments according to ANOVA and Tukey’s test (*p* < 0.05).

Upon the implementation of optimal conditions for buckwheat germ treatment, Figure 3 demonstrated a significant increase *(p* < 0.05) in the total flavonoid and phenolic contents of buckwheat sprouts subjected to UV-B treatment. Specifically, these contents attained levels that were 1.97 times and 1.54 times higher than those of the control group, respectively. Furthermore, it was clear that UV-B treatment suppresses the growth of buckwheat sprouts.

### 3.4. Effects of UV-B Treatment on Antioxidant System

**Figure 4:**
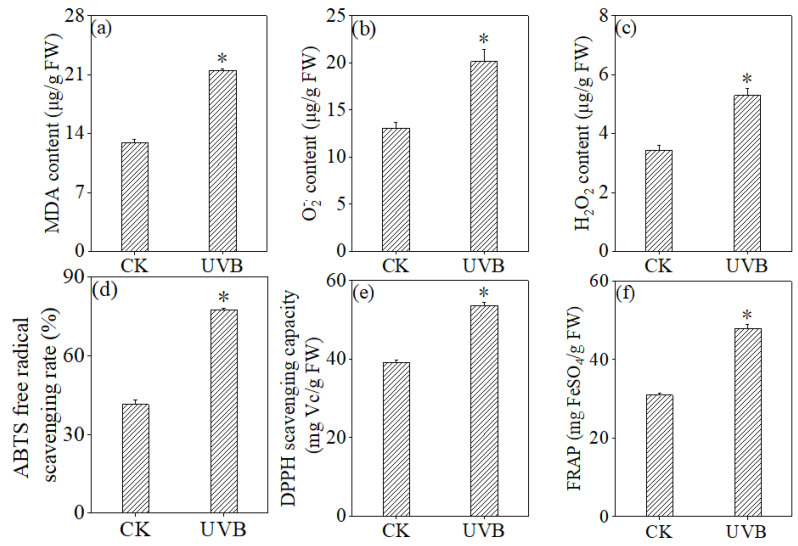
Effect of UV-B treatment on the content of MDA (**a**), $O_{2}^{-}$. (**b**), and H_2_O_2_ (**c**), ABTS free-radical scavenging rate (**d**), DPPH scavenging capacity (**e**), and FRAP (**f**). * Indicates significant differences in indicators among treatments according to ANOVA and Tukey’s test (*p* < 0.05).

In Figure 4, UV-B treatment significantly elevated the levels of MDA, H_2_O_2_, and $O_{2}^{-.}$ (*p* < 0.05), which were 1.66 times, 1.54 times, and 1.54 times greater than those in the control group, respectively. This result showed that UV-B treatment caused oxidative damage to buckwheat sprouts and induced oxidative stress in buckwheat sprouts.

Concurrently, UV-B treatment markedly elevated the ABTS free radical scavenging rate, DPPH scavenging capacity, and FRAP capacity of buckwheat sprouts (*p* < 0.05), which were 1.66 times, 1.54 times, and 1.54 times greater than those in the control group, respectively.

### 3.5. Effects of UV-B Treatment on Antioxidant Enzyme Activities and Expression Levels of Related Genes in Buckwheat Sprouts

**Figure 5:**
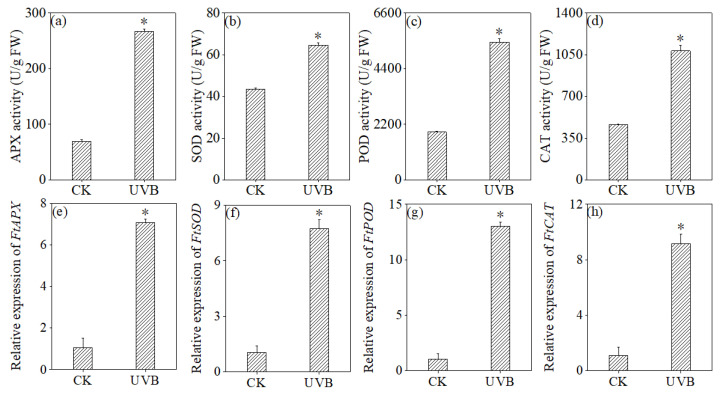
Effects of UV-B treatment on the activity of APX (**a**), SOD (**b**), POD (**c**), and CAT (**d**), and the relative expression of *FtAPX* (**e**), *FtSOD* (**f**), *FtPOD* (**g**), and *FtCAT* (**h**). * Indicates significant differences in indicators among treatments according to ANOVA and Tukey’s test (*p* < 0.05).

In Figure 5, UV-B treatment markedly increased the activity of the antioxidant enzymes (*p* < 0.05). This outcome aligns with the previously documented levels of antioxidant enzyme gene expression. The expression levels of the *FtAPX*, *FtCAT*, *FtPOD*, and *FtSOD* genes in buckwheat sprouts exposed to UV-B treatment were considerably elevated (*p* < 0.05). This discovery indicates that UV-B treatment can significantly enhance the antioxidant capabilities of buckwheat sprouts.

### 3.6. Effects of UV-B Treatment on the Expression Levels of Metabolic Enzymes and Related Genes

**Figure 6:**
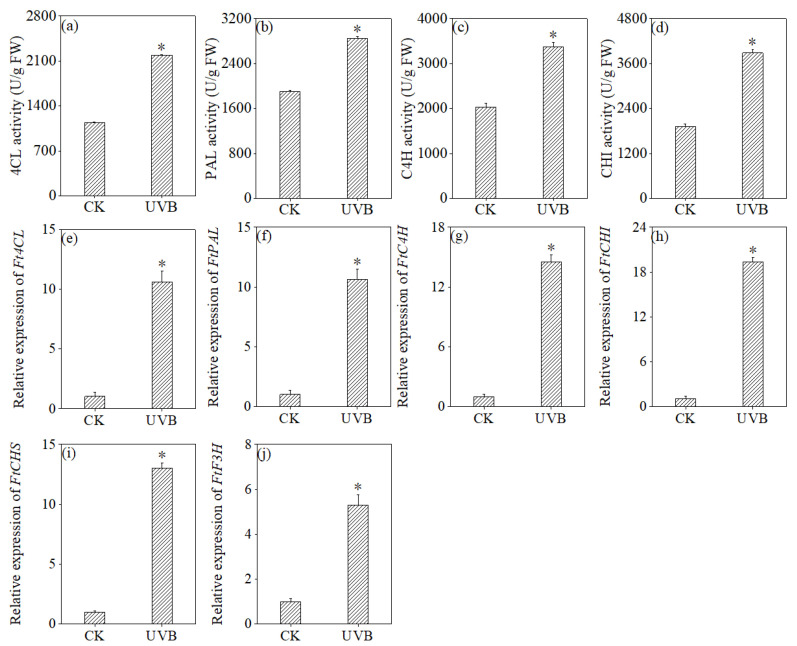
Effects of UV-B treatment on the activity of 4CL (**a**), PAL (**b**), C4H (**c**), and CHI (**d**), and the relative expression of *Ft4CL* (**e**), *FtPAL* (**f**), *FtC4H* (**g**), *FtCHI* (**h**), *FtCHS* (**i**) and *FtF3H* (**j**). * Indicates significant differences in indicators among treatments according to ANOVA and Tukey’s test (*p* < 0.05).

Figure 6 demonstrates that the UV-B treatment led to a statistically significant enhancement in the activities of metabolic enzymes, including 4CL, PAL, C4H, and CHI (*p* < 0.05), with increases of 1.91, 1.49, 1.66, and 2.02 times compared to the control group, respectively (*p* < 0.05). Moreover, the expression levels of genes related to these metabolic processes were significantly increased (*p* < 0.05). The results suggested that UV-B treatment can augment phenylpropanoid metabolism and promote metabolite accumulation.

## 4. Discussion

Buckwheat is a gluten-free, nutrient-rich crop with a high content of functional active ingredients, widely consumed in many countries, including those in Asia (such as China, India, and South Korea) and Europe (such as Ukraine, Poland, and Italy) [12]. However, the limitation of buckwheat consumption lies in its anti-nutritional components (such as protease inhibitors and tannins), which lead to relatively low bioavailability [16]. After buckwheat seeds germinate, not only can the content of anti-nutritional substances be reduced but the balance of protein and amino acid composition can also be improved, along with the digestibility and bioavailability of nutrients. More importantly, the accumulation of functional substances such as flavonoids and GABA can be further promoted [39,40]. Germinated buckwheat exhibits better antibacterial, antioxidant activity, and anti-obesity capabilities, while the quality of noodles and pancakes made from buckwheat sprouts has also been significantly improved [16,41].

Numerous studies have confirmed that UV treatment during the germination of plants such as soybeans [42], corn [43], and Finger Millet [28] is an effective method to promote the biosynthesis of secondary metabolites like flavonoids and phenolic compounds within the plants [44]. This is mainly because the accumulation of UV-absorbing compounds (primarily flavonoids) is one of the most important components of the plant’s repair and protection mechanism in response to UV radiation. Liu et al. [45] found that the biosynthesis of flavonoids is highly correlated with UV-B irradiation based on a summary of multi-species and multi-omics data from over 10,000 samples, showing the highest correlation. As a C3 crop that thrives in high-altitude areas with high levels of UV radiation, buckwheat also primarily responds to UV exposure by enhancing the accumulation of flavonoids in a dose-dependent manner [45]. However, the effects of UV irradiation conditions (such as UV dose, radiation quality, and exposure time) on the total flavonoid content in different buckwheat varieties vary. Henryk et al. [26] studied the effects of UV-B exposure on flavonoid content in four buckwheat varieties from Poland and found that the flavonoid content significantly increased in the Red Corolla and Kora varieties, showed no significant change in the Panda variety, and decreased in the Hruszowska variety. Yao et al. [25] investigated 15 buckwheat varieties grown under different natural UV-B exposure levels and similarly found differences in total flavonoid accumulation. In this study, the Sweet Buckwheat No. 3 variety was germinated under different intensities of UV-B irradiation, and significant differences in total flavonoid content were observed. Through single-factor and response surface experiments, the optimal UV-B irradiation intensity for accumulating isoflavones during germination of this variety was determined to be 30 μmol·m^−2^·s^−1^. Previous reports revealed that the optimal UV-B irradiation intensities for buckwheat [26] and soybeans [42] were 10 W/m^2^ and 10 µw/cm^2^, respectively. This further confirmed significant variability in plant species’ sensitivity to UV radiation. During buckwheat germination under UV exposure, flavonoid accumulation also varies. Earlier research indicated that flavonoid levels in buckwheat sprouts peaked on the 10th [46,47], 7th [23,48], and 6th [49] day, depending on the study. Our findings revealed a different pattern, with total flavonoid content rising initially and declining afterward, reaching its maximum on the third day. These discrepancies may stem from differences in varieties and germination conditions. Similarly, although previous studies optimized the germination temperature and showed that both temperature and time significantly influence flavonoid levels in buckwheat sprouts, germination temperature appeared to have a greater effect than germination time [30]. Our study also found that germination time, germination temperature, UV-B irradiation intensity, and duration all have significant effects on the flavonoid content in buckwheat sprouts, and there is an interaction between germination time and UV-B exposure time with UV-B irradiation intensity.

This study optimized the germination process using response surface methods to increase flavonoid levels in buckwheat sprouts through UV-B exposure. Under optimal conditions, the flavonoid content reached 1.97 times that of conventionally germinated sprouts, achieving 1872.84 μg/g FW. This finding aligns with previous research showing that UV treatment significantly boosts flavonoid accumulation in buckwheat [14,26]. The primary flavonoids identified include flavonols (e.g., rutin, quercetin-3-O-rhamnoside, vitexin, and quercetin-3-O-galactosyl-rhamnoside), flavones, and anthocyanins [19]. Notably, flavonoid concentrations in different buckwheat varieties and sprout parts followed distinct patterns under UV exposure [26,47]. UV irradiation also increased the proportion of anthocyanin-3-O-rhamnoside among total anthocyanins and led to cyanidin-3-O-rhamnoside accumulation in new tissues such as stems and leaves [50]. Furthermore, UV-treated sprouts synthesized flavonoids absent in grains, including oristin and vitexin [51]. This study primarily focused on total flavonoid changes during germination under UV-B, without examining variations in specific sprout parts or individual flavonoids. Future investigations could provide a more detailed understanding of UV effects on flavonoid composition in buckwheat sprouts.

Flavonoids belong to a highly complex and coordinated metabolic network, and their biosynthesis and regulation remain incompletely understood. The phenylpropanoid pathway in plants is recognized as a critical route for the accumulation of flavonoid secondary metabolites, where the activity of key enzymes and gene expression levels dictate their synthesis and buildup. Studies have identified a strong positive correlation between the activities of PAL [23], CHI, and α-galactosidase in buckwheat sprouts and total flavonoid content [22,24]. Treatments such as Ultraviolet-A, Ultraviolet-C, and their combination notably enhanced PAL, CHI, and rutin-degrading enzyme activity in buckwheat, thereby increasing flavonoid levels [21]. Similarly, blue light paired with UV-B exposure significantly boosted PAL and CHI activity [22]. In this study, UV-B treatment significantly elevated the activities of PAL, C4L, C4H, and CHI in buckwheat sprouts compared to control samples (Figure 6). This enhancement suggests that UV-B promotes total flavonoid accumulation by influencing the phenylpropanoid metabolic pathway. These findings align with previous reports on the effects of electromagnetic fields and mildly acidic electrolyzed water treatments [52] in inducing the phenylpropanoid pathway in buckwheat. Beyond enzyme activity, we analyzed the relative expression levels of six key genes, and UV-B irradiation led to notable increases in *FtPAL*, *FtC4H*, *Ft4CL*, *FtCHI*, *FtCHS*, and *FtF3H* expression after 3 days of germination (Figure 6). Other studies have demonstrated that germination [47], microwave exposure [53], and phenylalanine treatments [54,55] also significantly induced the expression of key genes such as *FtPAL*, *FtCHS, Ft4CL*, *FtF3H*, and *FtCHI* in buckwheat sprouts. Additionally, MYB transcription factors play a role in multiple metabolic pathways related to flavonoid biosynthesis and interact with signal response genes to regulate flavonoid synthesis and degradation [56]. However, the relationship between key gene transcription (*FtPAL*, *FtCHI*, and *FtFLS*) and MYB transcription factors (*FtMYB1*, *FtMYB2*, and *FtMYB3*) remains unclear and requires further study [16]. Additionally, proteomic analysis by Peng et al. [54] revealed that differentially abundant proteins in microwave-treated buckwheat sprouts are involved in flavonoid biosynthesis and phenylpropanoid pathways. Future research focusing on transcription factor regulation and the use of advanced omics technologies, including transcriptomics, proteomics, and metabolomics, can provide deeper insights into mechanisms driving flavonoid enrichment in buckwheat.

Although the total flavonoid and total phenol content significantly increased during the germination of buckwheat under UV-B irradiation, we still noted that the plants reached a compromise between the accumulation of flavonoids and biomass loss. This study observed that UV-B treatment inhibited buckwheat sprout growth, with significant increases in MDA, H_2_O_2_, and $O_{2}^{-.}$ contents, indicating ROS accumulation and lipid peroxidation-induced cell membrane damage (Figure 3 and Figure 4). UV-B exposure generally harms plant photosynthesis and DNA, slows growth and development, and reduces biomass. Jin et al. comprehensively examined the adverse impacts of UV-B on the survival and growth of species with varying life histories, nutritional conditions, and habitats [57]. For buckwheat, UV-B significantly decreased height, leaf area index, and biomass, leading to shorter plants and lower seed yields, with the extent of growth inhibition depending on the radiation dose [58]. However, through long-term evolution, buckwheat has developed a robust self-defense mechanism to counteract endogenous free radicals. Alongside changes in bioactive substances (such as flavonoids and phenols), the antioxidant defense system adjusts to withstand UV-B-induced stress. Our findings revealed that, compared to normal germination, UV-B exposure significantly enhanced the activities and gene expression levels of key antioxidant enzymes, including SOD, POD, CAT, and APX, in buckwheat sprouts (Figure 5). The combined effect of increased gene expression levels and enzyme activities of antioxidant enzymes help eliminate reactive oxygen species, thereby protecting plant cells from severe damage. This is consistent with previous research findings that soybeans [32,59], buckwheat [32,60], wheat [61,62], and Russian wildrye [63] respond to changes in the external environment by enhancing the activation of their antioxidant systems. Our study demonstrated that UV-B treatment significantly enhanced the antioxidant capacity of buckwheat sprouts, including ABTS and DPPH radical scavenging activity as well as FRAP capability. Similarly, previous research has reported that microwave [64,65], laser treatment [66], and trace element water treatment [67] effectively improved antioxidant properties in buckwheat sprouts. These findings suggest that regulating growth conditions to stimulate bioactive substances in buckwheat sprouts offers potential for exploring flavonoid enrichment mechanisms and developing innovative processes to boost flavonoid accumulation, thereby maximizing the functional value of buckwheat-based foods.

## 5. Conclusions

This study confirmed that buckwheat can effectively promote the biosynthesis of total flavonoids during germination under UV-B treatment. The optimal conditions for germination to maximize flavonoid accumulation were identified through single-factor and response surface experiments: buckwheat sprouts germinated for 3 days at 28.7 °C and exposed to UV-B radiation (30.0 μmol·m^−2^·s^−1^) for 7.6 h·d^−1^ during the germination period. UV-B treatment promoted total flavonoid synthesis and improved antioxidant capacity by boosting the activity and gene expression of key enzymes involved in the phenylpropanoid metabolism and antioxidant pathways. These buckwheat sprouts provide an excellent natural source of flavonoids and phenolic compounds, offering enhanced nutritional and antioxidant benefits. Flavonoid-enriched buckwheat sprouts have potential applications as ingredients in various nutritional products.

## Figures and Tables

**Table 1:** Regression model variance analysis.

Variables	Sum of Squares	d*f*	Means Square	F Value	*p* Value
Model	1.239 × 10^6^	14	88,484.46	26.70	<0.0001
A-germination time (d)	17,734.94	1	17,734.94	5.35	0.0364
B-UV-B treatment time (h·d^−1^)	1.12 × 10^5^	1	1.12 × 10^5^	33.69	<0.0001
C-germination temperature (°C)	22,536.56	1	22,536.56	6.80	0.0207
D-UV-B intensity (μmol·m^−2^·s^−1^)	19,048.20	1	19,048.20	5.75	0.0310
AB	2532.73	1	2532.73	0.76	0.3967
AC	5083.04	1	5083.04	1.53	0.2359
AD	15,829.53	1	15,829.53	4.78	0.0463
BC	2128.19	1	2128.19	0.64	0.4363
BD	49,405.73	1	49,405.73	14.91	0.0017
CD	1758.84	1	1758.84	0.53	0.4783
A^2^	4.85 × 10^5^	1	4.85 × 10^5^	146.36	<0.0001
B^2^	3.45 × 10^5^	1	3.45 × 10^5^	103.96	<0.0001
C^2^	4.74 × 10^5^	1	4.74 × 10^5^	143.01	<0.0001
D^2^	2.18 × 10^5^	1	2.18 × 10^5^	65.75	<0.0001
Residual	46,391.15	14	3313.65		
Lack of fit	40,318.41	10	4031.84	2.66	0.1796
Pure Error	6072.74	4	1518.19		
Cor total	1.29 × 10^6^	28			
*R*^2^ = 0.9639		*R*^2^_adj_ = 0.9278		CV = 4.03%	

## Data Availability

The original contributions presented in the study are included in the article/supplementary materials, further inquiries can be directed to the corresponding author.

## Supplementary Materials

The following supporting information can be downloaded at: https://www.mdpi.com/article/10.3390/foods13233928/s1, Table S1: Variables, their levels, and corresponding actual values utilized in the Box–Behnken design. Table S2: Box–Behnken design and datasheet. Table S3: Sequences of primers utilized in the study.
