# Supplementary material for: Optimization of Ultraviolet-B Treatment for Enrichment of Total Flavonoids in Buckwheat Sprouts Using Response Surface Methodology and Study on Its Metabolic Mechanism
Source: Foods. 2024 Dec 5;13(23):3928. doi: 10.3390/foods13233928 (PMC11641577; doi:10.3390/foods13233928)
Supplement: Supplementary file 1 [file foods-13-03928-s001.zip › foods-3346743-supplementary.pdf]

Table S1

Variables and their levels and actual values employed in Box-Behnken design

Independent variables	coding	levels		
		-1	0	1
germination time(d)	A	2	3	4
UV-B treatment time（h/d）	B	6	8	10
germination temperature (℃)	C	25	29	33
UV-B intensity（$\mu mol \cdot m^{-2} \cdot s^{-1}$）	D	25	30	35

Table S2

Box-behnken design and data sheet

No.	A<br>germination time (d)	B<br>UV-B treatment time (h/d)	C<br>germination temperature (°C)	D<br>UV-B intensity（μmol·m^{-2}·s^{-1}）	Y<br>flavonoid content (μg/g FW)
1	2	6	29	30	1392.36±46.54
2	4	6	29	30	1442.68±58.59
3	2	10	29	30	1283.32±70.00
4	4	10	29	30	1232.99±56.42
5	3	8	25	25	1434.30±69.11
6	3	8	33	25	1451.07±59.24
7	3	8	25	35	1367.20±67.23
8	3	8	33	35	1300.09±50.49
9	2	8	29	25	1342.03±45.85
10	4	8	29	25	1358.81±54.34
11	2	8	29	35	1459.46±32.41
12	4	8	29	35	1224.60±44.75
13	3	6	25	30	1459.46±48.54
14	3	10	25	30	1232.99±93.67
15	3	6	33	30	1417.52±58.59
16	3	10	33	30	1098.79±51.96
17	2	8	25	30	1459.46±57.39
18	4	8	25	30	1266.54±38.17
19	2	8	33	30	1241.38±15.19
20	4	8	33	30	1191.05±88.90
21	3	6	29	25	1442.68±32.15
22	3	10	29	25	1518.17±101.56
23	3	6	29	35	1543.34±67.00
24	3	10	29	35	1174.28±64.92
25	3	8	29	30	1786.58±27.88
26	3	8	29	30	1800.13±8.26
27	3	8	29	30	1836.25±28.16
28	3	8	29	30	1886.91±69.82
29	3	8	29	30	1820.13±81.67

Table S3
Primer sequences used in the study.

Primer	Sequences (5'-3')	
Actin	F[1] :TCGTGAGAAGATGACCCAGA	R[2] :CCGAGTCCAGCACAATACCT
PAL	F :TCTCCAGAAGCCGAAACAAG	R :AGCCTTGTTCCTGGATACAT
C4H	F :AACACACTACTCTCAGTTGC	R :ATTGGGTGATCGAGACTCTT
4CL	F :CTCTTTCACGTCCACGGTTT	R :GATGATTTGGTGGATGGTGG
CHS	F :CGTCAAGCGTTTCATGATGT	R :CAAGGCTTGTGTTGACATGG
CHI	F :ACTTTGAGGAATCCGCTGTGAC	R :AGGGCTTCAACATGGTGATCTGTA
F3H	F :CAAGGCTTGTGTTGACATGG	R :GACAGTGATCCAGGTCTTGC
CAT	F :GAGTTTGGTTCCCTTGCTT	R :TTCATACACTTCACTGGCGT
APX	F :GCTTCTCTTGAGCTTTGCTGT	R :TCTGTTGGGGAACACCGAGA
SOD	F :ATGGTGCTCCTGACGATG	R :CCACTGCCCTTCCAATAAT
POD	F :GTTCTGGTTGGGCTTGG	R :TTGTCCTCGTCTGTTGGTC

F[1]: Forward primer sequence, R[2]: Reverse primer sequence.
